# Extending the Storage Time of *Clanis bilineata tsingtauica* (Lepidoptera; Sphingidae) Eggs through Variable-Temperature Cold Storage

**DOI:** 10.3390/foods10112820

**Published:** 2021-11-16

**Authors:** Chenxu Zhu, Ming Zhao, Haibo Zhang, Fang Zhang, Yuzhou Du, Mingxing Lu

**Affiliations:** 1 College of Horticulture and Plant Protection & Institute of Applied Entomology, Yangzhou University, Yangzhou 225009, China; syzzzgalaxy@gmail.com (C.Z.); zhaoming@yzu.edu.cn (M.Z.); yzdu@yzu.edu.cn (Y.D.); 2 Plant Protection and Quarantine Station of Jiangsu Province, Nanjing 210009, China; zhanghbjs@126.com (H.Z.); cebaoke@126.com (F.Z.); 3 Joint International Research Laboratory of Agriculture and Agri-Product Safety, Yangzhou University, Yangzhou 225009, China

**Keywords:** *Clanis bilineata tsingtauica*, edible insects, cold storage, temperature, artificial breeding

## Abstract

*Clanis bilineata tsingtauica* Mell, 1922 (Lepidoptera, Sphingidae), also known as “Doudan” in China, is an important pest in legume crops. As an edible insect, it is most commonly consumed in Jiangsu, Shandong, and Henan Provinces. Mass rearing requires access to large numbers of eggs. This stage, however, is of short duration and supplies are frequently not sufficient for insect production. Therefore, we identified the cold storage conditions for *C. bilineata tsingtauica* that can effectively prolong the storage time of the eggs, to make supplies more readily available. We found that when stored at 4 °C, only 7.5% of the eggs hatched after 7 days, while at 10 °C the hatch rate was 78.3%. At 15 °C, the egg hatch rate remained at this same level (77.8% even after 14–20 days). Considering various combinations, we found that optimal egg hatch occurred if eggs were stored at 15 °C for 11 days, and then held at 15–20 °C under dark conditions. Stored as described above, the egg hatch rate was not significantly different from the control group (at 28 °C). These conditions allow for easier mass rearing of *C. bilineata tsingtauica* by providing a stable supply of eggs.

## 1. Introduction

Due to the increase in the world’s population, the need for proteins is increasing [1]. Due to limitations in the growth of the production of livestock, alternative meat products are of interest. In nutritional value, insects are comparable to traditional meat products and their production offers some advantages compared to increasing livestock production. Insects emit less greenhouse gas than vertebrate animals and the cost of insect rearing is lower than that of keeping vertebrates [1,2,3]. The interest in insects as food has increased ever since it was first suggested by Meyer-Rochow in 1975 that edible insects could ease the problem of global food shortages and that the FAO and WHO should support the use of insects as food and feed [3,4,5]. There are at least 2100 species of insects that are consumed in various parts of the world, especially species of beetles, crickets, caterpillars, bees, and wasps [3]. Insects may be fried, boiled, or processed as an ingredient added to flour, beer, candy, noodles, and protein bars [6,7,8,9,10,11].

**Figure 1:**
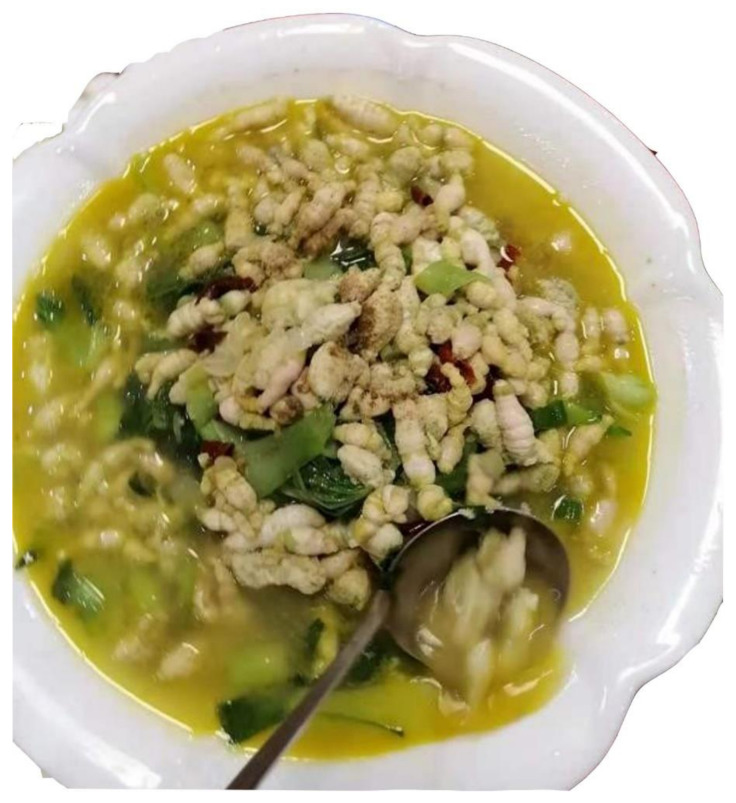
A common *Clanis bilineata tsingtauica* dish in a restaurant in Lianyungang City, Jiangsu Province.

*Clanis bilineata tsingtauica* Mell, 1922 (Lepidoptera, Sphingidae), in contrast to most edible insects, has a long history of consumption in China of 300 years plus [12]. *C. bilineata tsingtauica* larvae are rich in proteins, amino acids, fatty acids, vitamins, and trace elements, and they are a good source of nutrition [13,14,15,16]. *C. bilineata tsingtauica* is widely distributed in China. Breeding of *C. bilineata tsingtauica* has become an important source of income from agricultural activities for farmers in some areas of Jiangsu province. Currently, the market for *C. bilineata tsingtauica* products is gradually expanding, and has radiated to Shandong province, Nanjing city, Shanghai city, Guangzhou city, and other places in China. The annual output of *C. bilineata tsingtauica* through artificial breeding in the country is 3 × 10^4^ t, with an output value of nearly 4.5 billion RMB (0.7 billion USD). However, the annual demand for *C. bilineata tsingtauica* is about 10 × 10^4^ t, and the artificial breeding of *C. bilineata tsingtauica* is far from meeting people’s consumption needs [15]. Methods of cooking *C. bilineata tsingtauica* larvae include deep-frying, adding larvae into soups, cooking larvae together with vegetables, and stir frying them with chili (Figure 1).

*Clanis bilineata tsingtauica,* whose larvae are also known as “Doudan (豆丹)” in China, feed as larvae on legumes, for example soybeans, among other plants [17]. In some years, this species can be a pest of soybeans. In nature, *C. bilineata tsingtauica* has 1 or 2 generations per year in China. In Jiangsu Province, there is 1 generation per year. There are 5 instars, and the biomass increases dramatically in the 3rd and 4th instars. [18]. The weight of a 5th instar larva is around 8.15 g, and approximately 120 larvae weigh one kilogram. It is the 5th instar larvae that are usually sold in the market, and they are eaten in restaurants. To meet the demand for these larvae as human food, larvae are being reared in plastic greenhouses on soybean plants. The level of such production is expanding, and under ideal conditions three generations can be produced each year in Jiangsu Province.

For mass production of this insect, a crucial problem is the need for a reliable supply of high-quality eggs that can be synchronized with the production of soybean plants under greenhouse conditions. The duration of the egg stage of *C. bilineata tsingtauica* is between 3 and 5 days. Unless these eggs are held under suitable cold conditions, losses occur when trying to match the egg supply to development of the plant hosts. Meanwhile, due to the short duration of the egg stage, larvae may hatch during the long-distance transportation and cause losses. Thus, cold storage is an appropriate way of preserving *C. bilineata tsingtauica* eggs when there is a lack of host plants or transportation. Furthermore, considering the demand in the market, sometimes when supply exceeds demand, people can also cold store *C. bilineata tsingtauica* eggs and sell them when the market demand increases, which would lead to more profit.

For ectotherms, temperature is a major environmental factor that affects insect development and growth and almost all ecological and physiological processes [19,20,21,22,23,24]. Low temperature can harm insects being stored, causing reduced vitality or slow body metabolism [25,26,27]. Cold storage has been used to preserve and manipulate several stages of various insects, including eggs and pupae of *Chilo suppressalis* (Walker, 1863) (Lepidoptera, Crambidae) and eggs of *Corcyra cephalonica* (Stainton, 1866) (Lepidoptera, Pyralidae) and *Sitotroga cerealella* (Oliver, 1789) (Lepidoptera, Geechiidae) [27,28]. The desired temperature is one that will maintain the insects in a live and suitable stage, with adequate viability. For example, after cold-storing *Carposina sasakii* (Matsumura, 1900) (Lepidoptera, Carponisidae) eggs at 4 °C for 7 days, the hatch rate showed no significant difference from the control group [29]. The goal of this study was to determine the optimal temperature for cold storage of *Clanis bilineata tsingtauica* eggs to support mass-rearing facilities producing this species for human consumption. We developed and assessed a method of cold storage based on the use of variable storage temperatures, which we found to effectively prolong the storage time and retain egg viability.

## 2. Material and Methods

### 2.1. Source of Insect Materials

*Clanis bilineata tsingtauica* eggs were supplied from Suntiao Village, Yangji Town, Guanyun County, Jiangsu Province, China. *C. bilineata tsingtauica* larvae in our colony were reared on soybean leaves in glass containers.

### 2.2. Storage Container

The device for storing eggs consisted of a layer of moist filter paper on the bottom of a closed Petri dish to prevent the larvae hatched crawling away and affecting the statistics, with approximately 30 newly laid eggs scattered in the Petri dish. The Petri dish diameter is around 11.5 cm.

#### 2.2.1. Storage at Fixed Temperatures

Viability after storage was assessed for 4, 10, and 15 °C, where 4 °C and 10 °C are common temperatures in the cold storage of insects. Furthermore, 4 °C is the temperature of household refrigerators. It will be convenient if eggs could be stored at 4 °C with a high egg hatch rate and long storage time. Eggs were held at 4 and 10 °C for 7, 14, and 21 days in the dark all the time. The control group was held at 28 °C, which is the optimal development temperature for *C. bilineata tsingtauica*. Each treatment was replicated 4 times, with a replicate being one dish of 30 eggs.

Eggs were also held at 15 °C, which is the threshold for development [30]. Eggs held at 15 °C were kept in the dark until the last larva hatched. The control group was held at 28 °C. Each treatment (15 °C) and control group (28 °C) was replicated 3 times.

After storage, eggs were observed daily at the same time and any hatched or dead eggs were noted. A blackened or shriveled or empty egg indicates the death of the egg. The egg hatch rate, mortality rate, and stage duration were then calculated. Eggs were held under moist conditions to maintain humidity by spraying the filter paper with distilled water. Relative humidity in the test petri dishes and the incubator were held at 60−70%. Larvae from hatched eggs were reared at 28 ± 1 °C, 60−70% relative humidity, and L:D = 16:8 photoperiod, and were fed on soybean leaves in order to further observe the feeding intake and growth status. Larvae under different treatments consumed soybean leaves and molted regularly, as in the control group.

#### 2.2.2. Storage at Variable Temperatures

For this experiment, there were 5 treatments and 1 control, all with three replicates. The control group was held in the dark at 28 °C. This control group is the same as the one mentioned above when eggs were held at 15 °C. Before the experiment, eggs destined for the treatment groups were held in the dark at 20 °C for one day. Then, eggs were placed in the respective treatments in the dark, which were as follows:

Treatment 1: 15 °C for 5 days, 10 °C for 7 days, then put them under 28 °C
Treatment 2: 15 °C for 5 days, 10 °C for 7 days, then put them under 15 °C
Treatment 3: 15 °C for 10 days, 10 °C for 7 days, then put them under 28 °C
Treatment 4: 15 °C for 10 days, 10 °C for 7 days, then put them under 15 °C
Treatment 5: 15 °C for 11 days, then put them under 20 °C
Control: 28 °C all the time

**Figure 2:**
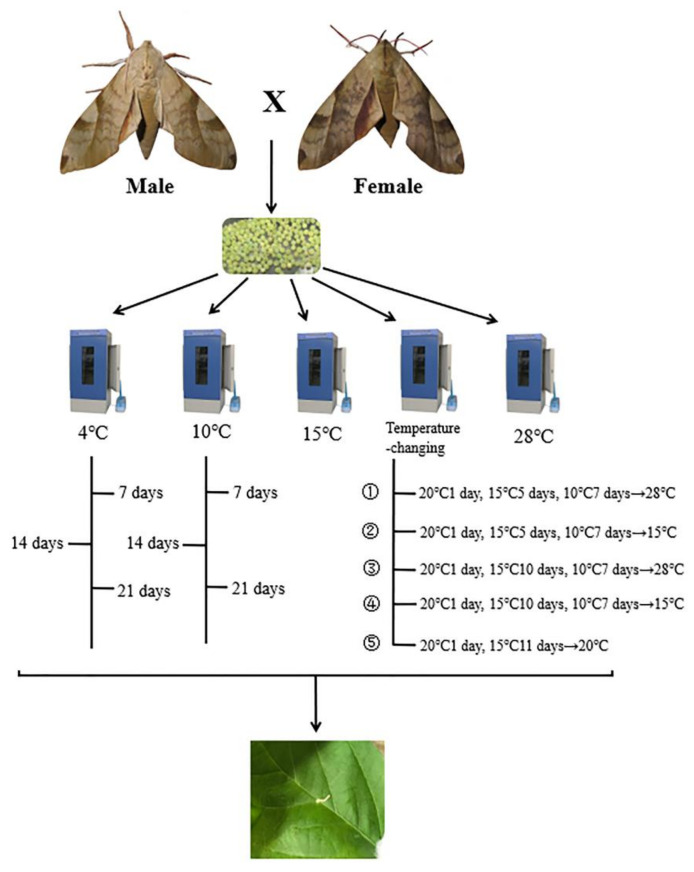
The routine of the whole experiment.

Egg hatch and egg mortality were observed daily at the same time and, from these data, we calculated the overall egg hatch and mortality rates for each treatment and the control. Eggs were held under moist conditions to maintain humidity by spraying the filter paper with distilled water. Relative humidity in the test Petri dishes and the incubator were held at 60−70%. When the larvae hatched, they were also observed and reared at 28 ± 1 °C, 60−70% relative humidity, and L:D = 16:8 photoperiod, and were fed on soybean leaves. Larvae under different treatments consumed soybean leaves and molted regularly, as in the control group. The routine of the whole experiment is showed below (Figure 2).

### 2.3. Statistical Analysis

The experimental data were analyzed by GraphPad Prism 8 and SPSS 16.0 (IBM Inc., New York, NY, USA). Significant differences were determined using the LSD (homogeneity of variances) or Dunnett T3 test (non-homogeneous). The hatch rates of eggs stored at 4 °C and 10 °C were analyzed using Dunnett T3. Student’s t-test (*p* < 0.05) was used to test whether the hatch rate of eggs stored at 15 °C was significantly different from the control group. The hatch rates of eggs stored at variable temperatures were analyzed using LSD. Dunnett T3 was used to analyze the storage time of eggs stored at 15 °C and variable temperatures.

## 3. Results

### 3.1. Effects of Storage at Fixed Temperatures on Egg Hatch

**Figure 3:**
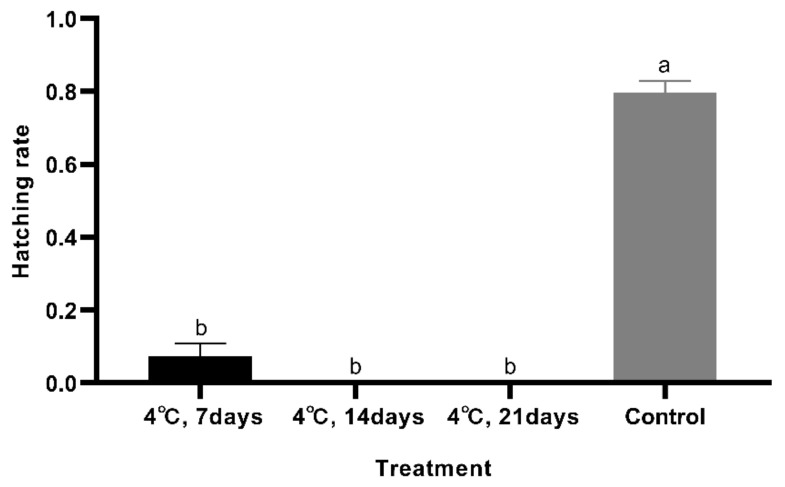
Difference analysis among the different treatments (4 °C). Bars represent the standard error. The hatch rates of eggs stored at 4 °C analyzed using Dunnett T3. Different letters on the bars indicate significant differences (*F*_3,12_ = 264.61; *p* < 0.05).

**Figure 4:**
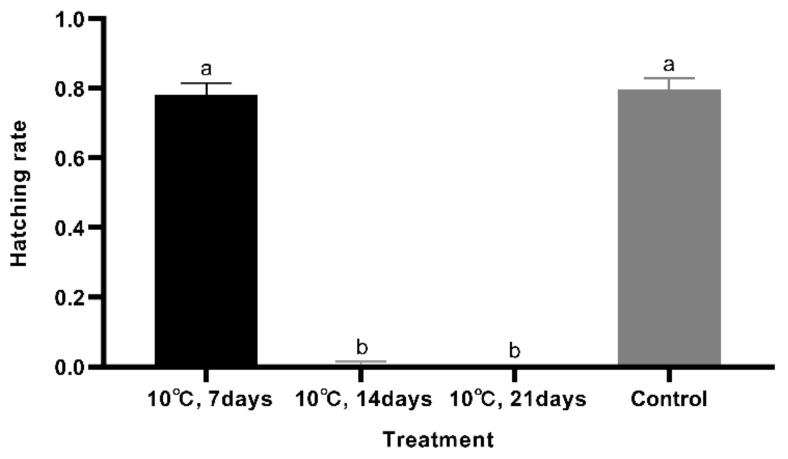
Difference analysis among the different treatments (10 °C). Bars represent the standard error. The hatch rates of eggs stored at 10 °C were analyzed using Dunnett T3. Different letters on the bars indicate significant differences (*F*_3,12_ = 378.74; *p* < 0.05).

**Figure 5:**
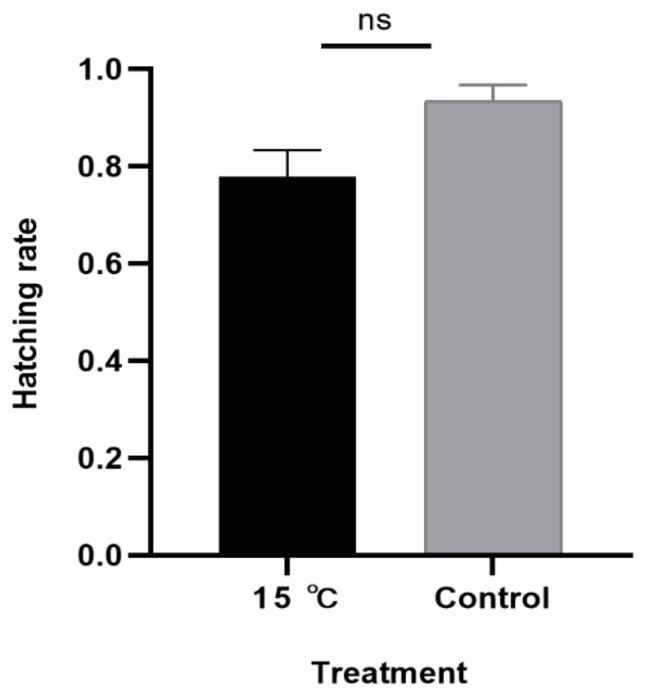
Difference analysis among the different treatments (15 °C). Bars represent the standard error. Student’s t-test (*p* < 0.05) was used to test whether the hatch rate of eggs stored at 15 °C was significant different from the control group (*p* = 0.074).

When eggs were stored at 4 °C for 7 days and then placed at 28 °C after storage, only 7.5% (±3.4%) (mean ± SE) of the eggs hatched. When eggs were stored at 4 °C for 14 days or longer, there was no hatch after storage. The hatch rate for the control group was 79.6% (±3.3%) (mean ± SE) (Figure 3). Eggs stored at 10 °C for 7 days and then returned to 28 °C had a hatch rate of 78.3% (±3.2%) (mean ± SE), which was not significantly different from the control (*p* = 1.000). When eggs were stored at 10 °C for 14 or 21 days, no eggs hatched after storage. (Figure 4). At 15 °C, 77.8% (±5.6%) (mean ± SE) of the eggs hatched, which was not different from the control group (*p* = 0.074) (Figure 5). Meanwhile, the feeding and growth status of the larvae from 15 °C were similar to the larvae from the control group. Therefore, 15 °C can be used as a temperature for cold storage.

### 3.2. Effects of Storage at Variable Temperatures on Egg Hatch

**Figure 6:**
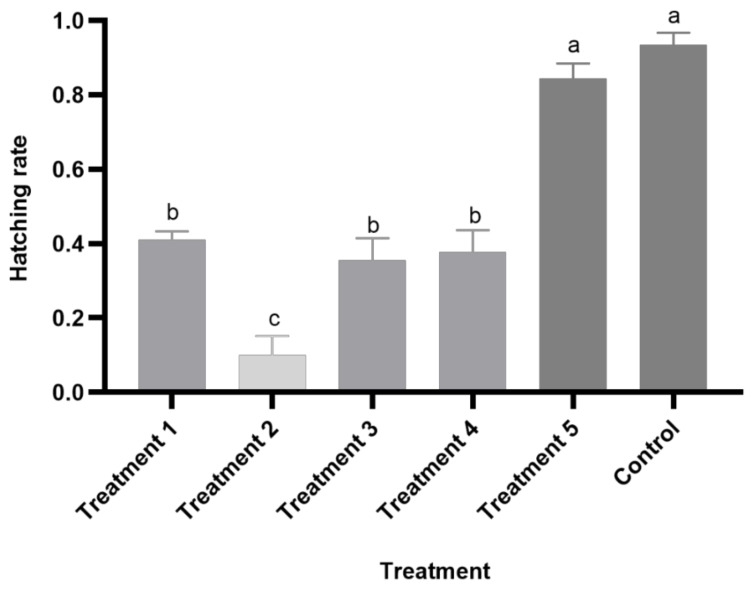
Effect of five variable temperature regimes on the hatching rate of *Clanis bilineata tsingtauica* eggs. Bars represent the standard error. The hatch rates of eggs stored at variable temperatures were analyzed using LSD. Different letters on the bars indicate significant differences (*F*_5,11_ = 44.43; *p* < 0.05).

Except for the fifth treatment, none of the treatments with variable combinations of colder and warmer temperatures produced eggs with hatch rates similar to the control (Figure 6). Only treatment 5, in which eggs were held at 15 °C for 11 days (just at the developmental threshold) and then brought up to 20 °C, had a high hatch rate (84.5% ± 4.0%) (mean ± SE), which exhibited a remarkable difference compared to the control hatch rate of 93.3% (±3.3%) (mean ± SE) (*p* = 0.216) (Figure 6). The feeding and growth status of the larvae from treatment 5 were not significantly different from the larvae from the control group.

### 3.3. Storage Times under Various Temperature Regimes

**Figure 7:**
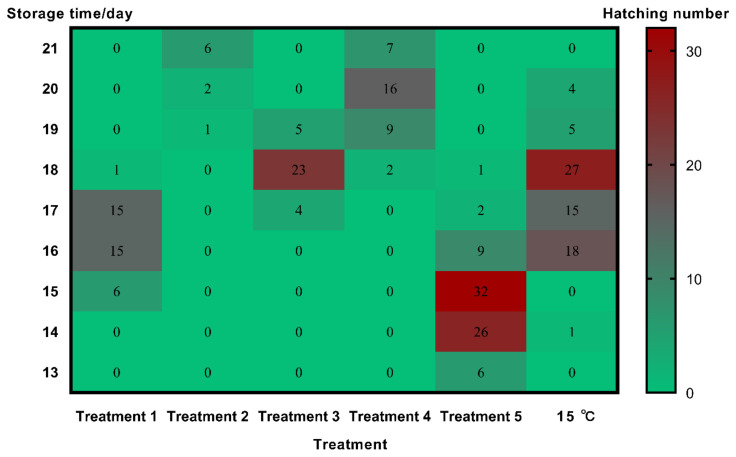
Heat map of the storage time and hatching number of each treatment.

**Figure 8:**
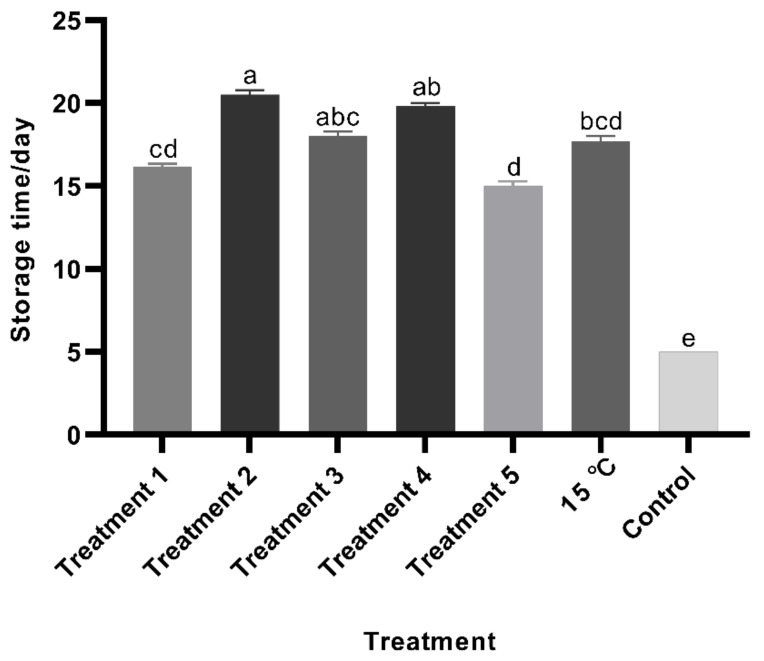
Storage times of *Clanis bilineata tsingtauica* eggs under different temperature regimes. Bars represent the standard error. Dunnett T3 was used to analyze the storage time of eggs stored at 15 °C and variable temperatures. Different letters on the bars indicate significant differences (*F*_6,14_ = 366.58, *p* < 0.05).

Storage time of the control group (at 28 °C) was only 5 days until hatching occurred. The storage times of treatments 1, 2, 3, and 4 were 15–18, 19–21, 17–19, and 18–21 days, respectively, but all of these treatments reduced the hatch rates to under 40% (Figure 7). The storage time of treatment 5 averaged 15.5 days but hatched similarly to the control. For eggs held continuously at 15 °C, the storage time averaged 17 days (Figure 8).

## 4. Discussion

*Clanis bilineata tsingtauica* is an edible insect with an expanding commercial market. As the demand for this product increases, the rearing scale will have to increase. Cold storage can be used to store *C. bilineata tsingtauica* eggs. We assessed various possible storage temperatures or combinations of temperature. Use of 4 °C for storage was examined because it is the temperature of household refrigerators and if suitable would be extremely convenient. However, from eggs stored at this temperature, almost none hatched, likely because a larva, not egg, is the overwintering stage in nature. The cold resistance of larvae is better than that of eggs and the developmental threshold temperature of larvae is lower than that of eggs [30]. At 10 °C, some larvae hatched when stored for 7 days; but, when stored longer at this temperature, the hatching rate declined. This phenomenon may be due to the fact that at low temperature eggs produce anti-cold biochemical substances to improve their cold tolerance [25,26,31], and with a longer storing time, the accumulation of cold-resistant substances reaches saturation. At this point they will consume a high fraction of metabolic energy to adapt to the external low-temperature environment. Even given sufficient and suitable growth conditions, they will not restore their original vitality. At 15 °C, which is the normal threshold temperature for egg developmental, many eggs hatched after a period of storage of 11 days. Combining this temperature with subsequent storage at 10 °C did not improve egg hatch. In general, storage at a temperature below the lower developmental threshold temperature lowered the hatch rate of eggs significantly. If eggs are held at a temperature above the developmental threshold temperature (15 °C), the ultimate hatching rate of the eggs is not harmed, and the storage time can be prolonged for over 2 weeks. In the same time, the feeding and growth status of the larvae were similar to those of the control group. This finding is of great importance to mass reproduction systems and will allow eggs to be held until needed over a period of several weeks between production and use. Being able to store eggs when market demand for them is low or during the transportation can help increase profitability and make possible shipping eggs between different regions. The storage time, however, is not long (14–20 days), and future work on selection of eggs best adapted to longer storage should be pursued.

Alternatively, markets could use larvae rather than eggs as the stage to initiate rearing, given that *C. bilineata tsingtauica* overwinters as diapausing larvae [30]. However, breaking of the larval diapause after having received the material would take time, which would be disadvantageous in mass-rearing operations; also, even after diapause termination, the growth and feeding of the resulting larvae may be affected [32], or larvae may remain in quiescence for several months [33]. In general, technology for effectively breaking diapause in insects is not well developed. However, if methods to break diapause effectively could be developed, it would solve a major problem limiting the mass reproduction of this insect and likely would be valuable for the cold storage of other insects as well.
